# BCG-Induced Cross-Protection and Development of Trained Immunity: Implication for Vaccine Design

**DOI:** 10.3389/fimmu.2019.02806

**Published:** 2019-11-29

**Authors:** Camila Covián, Ayleen Fernández-Fierro, Angello Retamal-Díaz, Fabián E. Díaz, Abel E. Vasquez, Margarita K. Lay, Claudia A. Riedel, Pablo A. González, Susan M. Bueno, Alexis M. Kalergis

**Affiliations:** ^1^Millennium Institute on Immunology and Immunotherapy, Departamento de Genética Molecular y Microbiología, Facultad de Ciencias Biológicas, Pontificia Universidad Católica de Chile, Santiago, Chile; ^2^Departamento de Biotecnología, Facultad de Ciencias del Mar y Recursos Biológicos, Universidad de Antofagasta, Antofagasta, Chile; ^3^Sección de Biotecnología, Instituto de Salud Pública de Chile, Santiago, Chile; ^4^Facultad de Medicina y Ciencia, Universidad San Sebastián, Providencia, Santiago, Chile; ^5^Millennium Institute on Immunology and Immunotherapy, Departamento de Ciencias Biológicas, Facultad de Ciencias de la Vida, Universidad Andrés Bello, Santiago, Chile; ^6^Departamento de Endocrinología, Escuela de Medicina, Facultad de Medicina, Pontificia Universidad Católica de Chile, Santiago, Chile

**Keywords:** BCG, innate immunity, trained immunity, heterologous protection, vaccine

## Abstract

The Bacillus Calmette-Guérin (BCG) is a live attenuated tuberculosis vaccine that has the ability to induce non-specific cross-protection against pathogens that might be unrelated to the target disease. Vaccination with BCG reduces mortality in newborns and induces an improved innate immune response against microorganisms other than *Mycobacterium tuberculosis*, such as *Candida albicans* and *Staphylococcus aureus*. Innate immune cells, including monocytes and natural killer (NK) cells, contribute to this non-specific immune protection in a way that is independent of memory T or B cells. This phenomenon associated with a memory-like response in innate immune cells is known as “trained immunity.” Epigenetic reprogramming through histone modification in the regulatory elements of particular genes has been reported as one of the mechanisms associated with the induction of trained immunity in both, humans and mice. Indeed, it has been shown that BCG vaccination induces changes in the methylation pattern of histones associated with specific genes in circulating monocytes leading to a “trained” state. Importantly, these modifications can lead to the expression and/or repression of genes that are related to increased protection against secondary infections after vaccination, with improved pathogen recognition and faster inflammatory responses. In this review, we discuss BCG-induced cross-protection and acquisition of trained immunity and potential heterologous effects of recombinant BCG vaccines.

## Introduction

One of the leading causes of human death worldwide is tuberculosis (TB), a bacterial infection caused by *Mycobacterium (M.) tuberculosis*. In 2017, 10 million people developed TB disease, causing 1.3 million deaths (1). To prevent TB, a vaccine was developed in 1921 by Albert Calmette and Camille Guérin, which is currently included in the immunization programs of most countries. This vaccine consists of an attenuated *M. bovis* bacillus that was repeatedly passaged in culture by Calmette and Guérin and which is known as the bacillus Calmette-Guérin (BCG) (2). This vaccine was developed from a virulent *M. bovis* strain that accumulated more than 14 genome deletions in different regions (3). In most countries, BCG is administered to newborns a few hours or days after birth and its protective effects against tuberculous meningitis and miliary tuberculosis (TB) have shown efficacy over 70%, while its protective effect against pulmonary TB displays an average of 52% of protection (4–7). Mathematical estimations suggest that BCG vaccination of 100.5 million children, out of the 132.8 million children born in the world, prevented nearly 30,000 cases of TB meningitis and 11,500 cases of miliary TB in the year 2002 (6). In adults, BCG vaccination fails to completely protect against pulmonary TB, showing a range of effectiveness between 0 and 80% (8–10), which explains why TB is one of the major causes of mortality worldwide (1). Despite this, in 2018 BCG was considered within the national vaccination program of 154 countries, including countries in America, Asia, Africa, and Europe, with coverage of over 90% (1). It was also administered to high-risk groups in additional countries, being one of the most widely used vaccines worldwide (1, 11). Besides protecting against TB, BCG vaccination also reduces mortality in children because of non-specific cross-protection induced by this vaccine against other unrelated pathogens (12, 13). Initial evidence for this phenomenon was described in Sweden in 1927 by the physician Carl Näslund, who found that during the first year of life, BCG-vaccinated newborns had a mortality rate that was three times lower than unvaccinated babies (14). This observation was also made by Albert Calmette, in 1931 (15). In Guinea-Bissau, a country with a high childhood mortality rate, the presence of a BCG-vaccination scar was associated with diminished mortality rates associated with malaria or unclassified fever (16). Besides, BCG-vaccinated children showed a reduced risk of developing acute lower respiratory tract infections (ALRI) as compared with non-vaccinated ones (17). Furthermore, several studies carried out in West Africa showed over a 40% reduction in mortality after BCG vaccination, preventing malaria, sepsis, respiratory infections, and leprosy (14, 16, 18–21). In Spain, BCG vaccination reduced hospitalizations due to respiratory infections unrelated to TB in children under 14 years of age (13). Also, reduced child mortality due to BCG vaccination has been observed in other places of the world, including Sweden, United Kingdom, South or Southeast Asia, India, and Haiti (22–24).

Another remarkable characteristic of BCG is that it can be used as an expression vector for recombinant antigens to develop novel vaccines for pathogenic bacteria and viruses (25–34), as well as for cancer diseases (35–43). BCG has been considered as a good vector given its safety shown in vaccinated neonates, children and adults for almost a 100 years and that BCG antigens may act as adjuvants, inducing innate and adaptive immune responses (11, 22–24, 44, 45).

## Immune Response Induced by BCG Vaccination

The immune response elicited after BCG vaccination begins at the inoculation site after intradermal injection, where resident neutrophils, macrophages, and dendritic cells (DCs) interact with the bacillus (44, 46). The recognition of BCG by immune cells takes place through the interaction of different pattern recognition receptors (PRRs) with pathogen-associated molecular patterns (PAMPs), such as peptidoglycan, arabinogalactan, and mycolic acids located at the bacterium cell wall (44). Among the receptors involved in the recognition of BCG are toll-like receptors (TLRs) TLR2 and TLR4 present on the cell surface membrane (44). It has been shown that different proteins expressed by *mycobacteria* can work as TLR agonists, stimulating macrophage, and DC maturation and the secretion of pro-inflammatory cytokines (47). Likewise, complement receptors CR3 and CR4 are involved in the recognition of opsonized *mycobacteria* by DCs. Another group of cell receptors that recognize BCG PAMPs are nucleotide-binding oligomerization domain (NOD)-like receptors found in the cytosol of innate immune cells, such as NOD2, which interact with a specific component of the bacterial peptidoglycan (48). Besides, C-type lectins, such as DC-specific intercellular adhesion molecule-3-grabbing nonintegrin (DC-SIGN) interact with components of the bacterial wall and are involved in the recognition and internalization of BCG (48). After internalization by DCs, the mycobacterium can live up to 2 weeks inside these cells (49). This interaction induces DC maturation and migration that is characterized by an increase in the expression of co-stimulatory molecules, such as CD40, CD80, CD83, and CD86 (50). One of the antigens present in the cell wall of BCG corresponds to antigen (Ag) 85 (also present in *M. tuberculosis*), which stimulates the production of tumor necrosis factor-alpha (TNF-α), interleukin 1-beta (IL-1β) and IL-6 (51, 52), which are able to generate an pro-inflammatory state that promotes the activation of immune cells (50).

**Figure 1:**
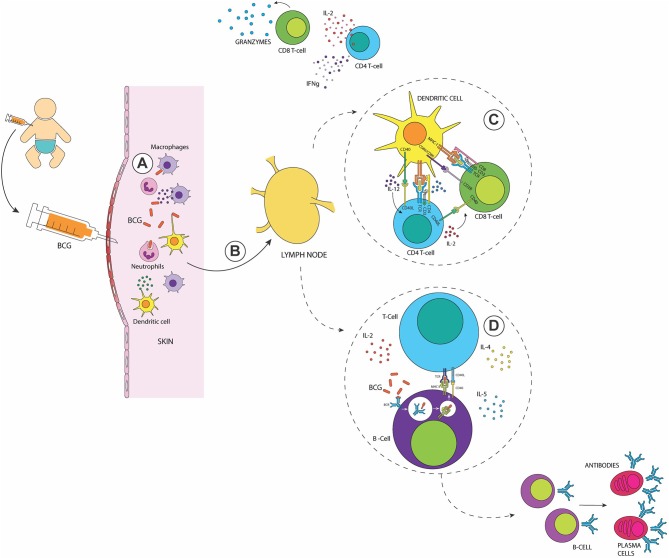
The immune response elicited after BCG vaccination in the newborn. **(A)** Recognition of the BCG at the inoculation site by neutrophils, macrophages, and DCs. **(B)** Activated skin DCs migrate to the draining lymph nodes to activate adaptive immune cells **(C)** Activation of Mycobacteria-specific CD4^+^ and CD8^+^ T cells with a T_H1_ profile, secreting elevated amounts of IFN-γ and granzymes **(D)** Activation of B cells leads to the generation of memory and plasma cells and the production of antigen-specific antibodies in response to the presence of antigens of BCG. After their activation, memory T and B cells reside in lymph nodes.

The development of an adaptive immune response starts when antigen-presenting cells (APCs, e.g., DCs, macrophages and B cells) present antigenic peptides on MHC molecules and prime T cells located at the nearest secondary lymphoid tissues or the spleen (53). *In vitro* and *in vivo* studies have shown that BCG-infected skin DCs migrate to the draining lymph nodes where they secrete TNF-α, IL-6, and IL-12 and activate both, CD4^+^ and CD8^+^ T cells (54–57) (Figure 1). Interestingly, it has been reported that BCG-infected human neutrophils cooperate with infected DCs to stimulate antigen-specific T cell responses (58).

As summarized in Figure 1, the adaptive immune response induced after BCG vaccination involves the activation of both, CD4^+^ and CD8^+^ T cells (53, 59) with elevated production of IFN-γ, which increases the anti-mycobacterial activity of macrophages (52, 53). This cytokine also contributes to the activation of B cells and the subsequent generation of antigen-specific antibodies by plasma cells. In early stages after vaccination, a pool of mycobacteria-specific CD8^+^ T cells proliferates and is present in peripheral blood up to 10 weeks after BCG vaccination (60). These CD8^+^ T cells were able to secrete IFN-γ and express granzymes, as well as perforins supporting the cytotoxic potential for these cells (60, 61). Activated T_H1_ CD4^+^ T cells have also been detected (62, 63), which produce large amounts of IFN-γ, TNF-α, and IL-2 (55, 64). In newborns, BCG-specific CD4^+^ T cells could be detected in the peripheral blood 3 weeks after vaccination, with a peak at 10 weeks (60). Studies with T cells transferred from BCG-vaccinated mice into animals that are deficient for both B and T cells have shown that CD4^+^/CD8^+^ T cells are necessary for reducing and controlling bacterial dissemination (65). During the contraction phase, BCG-specific CD4^+^ and CD8^+^ T cells switch to a memory phenotype, with functional features of effector memory T cells secreting IFN-γ (64, 66). These memory T cells generate a strong lymphoproliferative response to TB antigens several months after vaccination in mice (66).

Between 4 and 8 weeks after BCG vaccination, there is an induction of a B cell response that increases the production of IgG (67) and induces long-lived memory B cells (68). These IgG molecules can opsonize BCG and *M. tuberculosis*, enhancing phagocytosis and the inhibition of intracellular bacterium growth (67). It was shown that mucosal BCG vaccination induces an airway-resident memory T cell population in the lungs, but immunoglobulin production was not measured in this study (69). Pulmonary immune response in mice infected with *M. tuberculosis* was improved when BCG was administered intranasally as compared to subcutaneous vaccination (70). This improved protective efficacy was concordant with an increased presence of IgA and CD4^+^IL-17A^+^ T cells in bronchoalveolar lavages of intranasally vaccinated mice (70). Although subcutaneous BCG vaccination enhances blood IgG levels, in the case of respiratory airway pathogens, IgA confers better protection against infections (70). This is due to the fact that IgA is constitutively found in serum and mucosa and corresponds to one of the first barriers of defense (71). These antibodies can neutralize, excrete pathogens and activate the immune response by the modulation of the secretion of cytokines, such as TNF-α and IL-1β (71).

## BCG Vaccine As a Strategy for Modulating Immunity

**Figure 2:**
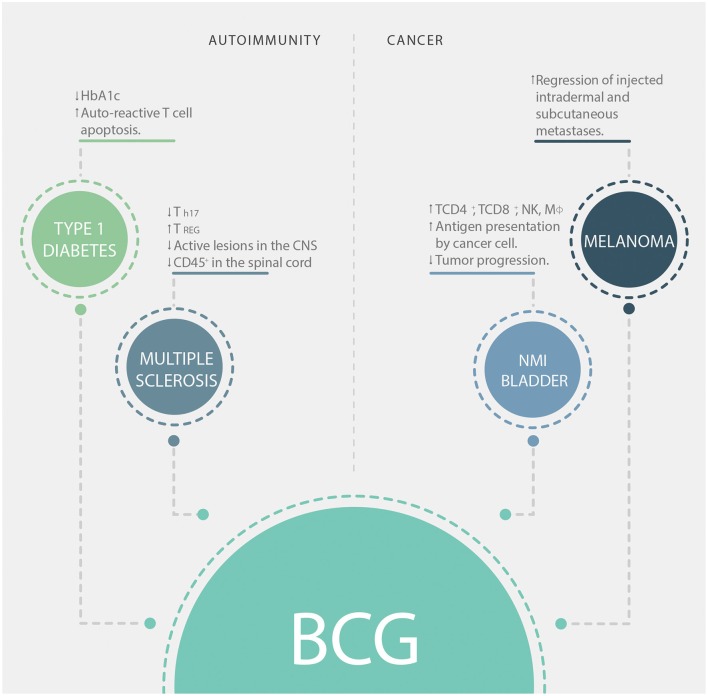
BCG applications in immunotherapy. Clinical applications of BCG in autoimmunity (left boxes) and cancer (right boxes). HbA1c, glycosylated hemoglobin; CNS, central nervous system; NK, natural killer cells; Mϕ, macrophages; NMI, non-muscle invasive.

Besides protection against TB, BCG has other clinical applications, especially in two main immunotherapy fields: treatments for cancer and autoimmune diseases, such as melanoma and type-1 diabetes (T1D), respectively (we summarize the contribution of BCG to immunotherapy in Figure 2). T1D is an autoimmune disease characterized by the destruction of pancreatic beta cells (72). This destruction leads to a lack of insulin production, which leads to the development of hyperglycemia, polyuria, and hypoinsulinemia (73). The effect of BCG vaccination in T1D is still controversial, as it was originally observed that BCG vaccination promoted a remission of the disease when patients were treated during the first month after diagnosis (74). On the other hand, a randomized clinical trial performed in 1999 where patients between 5 and 18 years old were vaccinated early after disease appearance showed no difference in glycated hemoglobin levels (HbA1c, an indicator of blood glucose levels) or endogenous insulin secretion compared to non-vaccinated ones (75). In a phase I trial performed in adults with long-term T1D, BCG vaccination in multiple doses was able to reduce HbA1c levels and increased the death of insulin-autoreactive T cells (76). Interestingly, BCG modulation of blood sugar was associated with a systemic shift toward a glycolytic pathway of glucose utilization (76). Another clinical trial performed with long-term T1D patients showed that vaccination with BCG stabilized HbA1c levels without producing hypoglycemia, an effect that could last up to 8 years after vaccination (77). In non-obese diabetic (NOD) mice, injection of BCG was shown to reduce insulitis and diabetes development (78). It has been demonstrated that the stimulation of TNF-α induced by BCG is involved in the destruction of insulin-autoreactive T cells (79). Despite this, the mechanism involved in the immunomodulatory effect induced by BCG in T1D patients has not yet been elucidated.

Another important autoimmune disease is multiple sclerosis (MS), which is characterized by the development of neurological symptoms due to gradual demyelination of the central nervous system (CNS) as a consequence of an inflammatory-autoimmune response (80). At present, a specific treatment for this disease that is effective is not available. Nonetheless, clinical trials showed that BCG vaccination could reduce the frequency of active lesions in the CNS of 12 MS patients (81). Further, a phase II clinical trial showed that after the first demyelinating episode, BCG vaccination reduced the risk of developing clinically definite MS for 5 years (82). Moreover, in a widely used mouse model of MS named experimental autoimmune encephalomyelitis (EAE), the injection of subcutaneous extended freeze-dried (EFD) BCG attenuated the severity of EAE (83). Mice treated with EFD BCG showed significantly lower clinical scores and reduced infiltration of CD45^+^ cells in the spinal cords (83). Furthermore, this treatment was shown to reduce the frequency of T_H17_ cells and to increase the frequency of T_REG_ cells in secondary lymph nodes. This consequence will help to limit the inflammation induced by EFD BCG in EAE (83).

Since several studies have demonstrated that BCG induces T_H1_/T_H17_ responses against TB and other unrelated pathogens (62, 84, 85), its capacity to exert a regulatory effect over autoimmune diseases, such as T1D and MS is very surprising. However, there are immune-metabolic pathways involved in the activation of the immune system after BCG vaccination that may account for explanations of these observations. Indeed, the activation of innate immune cells and T cells induced by this vaccine is partly mediated by the activation of cell glycolytic pathways (86). Besides, human Treg cells are highly glycolytic (87). Based on these findings, Ristori et al. proposed in 2018 that BCG induces a tolerogenic response via enhancement of glycolysis, contributing to the reduction of inflammation in autoimmune diseases (88). Another possible mechanism through which BCG can mediate protection in the context of autoimmune diseases relays on the immune response to the infection with the mycobacterium. After infection, it has been shown that activated, but not naïve, CD4^+^ T cells undergo apoptosis in an IFN-γ-dependent manner (89). Thus, apoptosis of activated T cells may have as a consequence the diminution of activated autoreactive cells, improving the health condition of the individual receiving vaccination. Also, TLR-signaling stimulated by mycobacterial components induces IL-10 secretion by B cells and consequent suppression of Th1 and Th17 activities, contributing to the suppression of autoimmune reactions (90).

On the other hand, there is increasing evidence for the use of BCG vaccination for the prevention and treatment of cancer (35–43). Vaccination of newborns with BCG reduces the risk of developing melanoma (35) and childhood leukemia (36). In the case of melanoma, direct vaccination with BCG into nodules of intradermal or subcutaneous metastases induced their regression in 90% of the injected lesions (37). As previously described, BCG exposure of tumoral macrophages induces transcriptional reprogramming of these cells, leading to an improved pro-inflammatory phenotype (91). BCG-treated macrophages can induce the activation of T cells infiltrating the tumor, thus improving antitumor immunity (91). Moreover, in patients with non-muscle invasive bladder cancer, it has been shown that BCG instillation after transurethral resection reduces tumor progression (38, 92). BCG impairs the tolerogenic milieu developed by carcinogenic cells, inducing local infiltration of macrophages, CD4^+^ T helper cells, CD8^+^ T cells, and NK cells, resulting in the development of local inflammation (40, 41, 93). Bladder tumor cells express antigen-presenting and co-stimulatory molecules after being infected with BCG, suggesting that these cells can function as antigen-presenting cells making them a good target to be destroyed by cytotoxic cells (42). The antitumor effects of BCG immunization could also be associated with the development of local inflammation that might overcome the tolerogenic environment induced by tumor cells (43).

The different effects observed after the administration of the BCG vaccine suggest that the activation of the immune system induced by it could vary depending on the environment in which it is found. BCG administration could exert a beneficial modulation of the immune system in the context of autoimmune diseases due to a redirection of the inflammatory response. In the case of cancer, the activation of the immune system in the presence of the bacteria within the tumor alters the tolerogenic environment induced by cancer cells, leading to a specific cytotoxic activity against the tumor cells.

## Trained Immunity As a Consequence of BCG Vaccination

Netea et al. (94) were the first to propose the concept of “trained immunity,” which is defined as an increased non-specific response to a secondary infection mediated by the innate immune system, either to the same or different microorganisms (94). This type of immunity is characterized as being independent of T and B cell responses and is mediated by monocytes/macrophages and NK cells (95).

In humans, BCG vaccination of adults induces a trained phenotype in circulating monocytes, characterized by an increased capacity to produce proinflammatory cytokines, an effect that has translated to non-specific protection against unrelated pathogens, such as *S. aureus* and *C. albicans* (96, 97). Also, BCG vaccination of healthy human volunteers increased the capacity of NK cells to secrete proinflammatory cytokines, such as IL-1β and IL-6 after stimulation with *M. tuberculosis* or unrelated pathogens (*S. aureus, C. albicans*) (98). These observations were performed 3 months after vaccination, consistent with the fact that BCG reduces mortality in newborns during the first year of life, as mentioned above. Thus, BCG induces non-specific protection against unrelated pathogens (96).

Interestingly, innate immune cells mediate this nonspecific protection, independent of T and B cells. A comparison of systemic lethal candidiasis infection in severe combined immunodeficiency (SCID) mice, which lack T and B cells, and NOD/SCID/IL2Rγ (NSG) mice that lack T, B and, NK cells, showed that partial protection was mediated by NK cells in BCG-vaccinated mice (98). Specifically, mice were challenged with a lethal intravenous dose of *C. albicans* 2 weeks after BCG vaccination; while SCID vaccinated mice survived, NSG vaccinated mice were partially protected, suggesting a role for NK cells in the non-specific protective effect induced by BCG vaccination (98).

Besides, there is *in vitro* and *in vivo* evidence of BCG-related trained immunity effects in bovine monocytes (99). *In vitro* exposure of calf monocytes to BCG leads to enhanced TNF-α and IL-6 production after subsequent TLR agonist stimulation. Aerosol BCG vaccination of calves exerted a similar effect of PBMCs, boosting pro-inflammatory cytokine production, in association with a shift to anaerobic glycolysis (99). The trained immunity phenotype was also observed testing PBMCs obtained 3 months after vaccination (99), which further supports previous studies carried out in human cells.

It has been shown that one of the molecular mechanisms that induces the development of trained immunity is epigenetic reprogramming, specifically through histone modifications (95). Epigenetic modifications regulate gene expression in response to environmental signals (100). In the immune system, epigenetic modifications are involved in cell differentiation, inflammation and autoimmune diseases (96, 100–103). Different types of epigenetic modifications have been described, including DNA modifications, non-coding RNAs, histone modifications and chromatin remodeling (100). Histone modifications are highly dynamic and can change within minutes; there are different classes of modifications such as acetylation, methylation, phosphorylation, ubiquitylation, sumoylation, ADP ribosylation, deimination, and proline isomerization (104). Histones can be methylated at arginine or lysine residues. Lysine can accept up to three methyl groups, being mono-, di- or trimethylated. Arginine can be mono- or dimethylated (105). These modifications are involved in the activation or repression of the transcription of genes that they are associated with. While methylation of lysine 4 in histone 3 (H3K4), H3K36, and H3K79 are usually associated with transcription activation; methylation in H3K9, H3K27, and H4K20 are associated with gene silencing (105).

After BCG vaccination, peripheral blood monocytes show an increase in H3K4me3 histone modification associated with the promoters of the genes *tnf*α*, il6*, and *tlr4* that lead to the transcriptional activation of these proinflammatory cytokines (96, 106). These responses are dependent on the nucleotide-binding oligomerization domain 2 (NOD2) receptor present in monocytes and receptor-interacting protein kinase 2 (Rip2) (96). These epigenetic modifications upregulated the expression of pattern recognition receptors (PPRs), namely TLRs, C-type lectins receptors, NOD-like receptors, and RIG-I-helicases that specifically recognize pathogen-associated molecular patterns (PAMPs) and modulated the accessibility of transcription factors to proinflammatory cytokine genes (96). Consequently, when these trained monocytes are exposed to a second infection, the pathogen is recognized by PPRs, leading to increased cytokine production (95).

In addition to epigenetic reprogramming, different cellular metabolic pathways are involved in the regulation and development of trained immunity in monocytes, macrophages and NK cells (86, 107–109). Indeed, glycolysis metabolism is increased in human monocytes after BCG vaccination, leading to a shift in the metabolic programming of the cell from oxidative phosphorylation to aerobic glycolysis (Warburg effect) (108). Additionally, it has been demonstrated that inhibition of the glycolytic pathway impairs the development of a trained immunity phenotype by preventing epigenetic rearrangements (86). Specifically, it was shown that glycolysis inhibits epigenetic modifications at the promoters of genes encoding for IL-6 and TNF-α in peripheral monocytes (86). Glutaminolysis and cholesterol synthesis have also been involved in the development of trained immunity in monocytes, being fumarate a key metabolite that can induce chromatin rearrangements. This metabolite induces an increase in H3K4me3 in the promoters of *tnfa* and *il6* leading to elevated secretion of these cytokines upon re-stimulation with LPS (107). Additionally, accumulation of mevalonate, a metabolite of the cholesterol synthesis pathway, has also been shown to be able to induce trained immunity through the enrichment of H3K4me3 in the promoters of *tnfa* and *il6* (109). All these reports support the notion that epigenetic regulation is intimately related and coordinated with the metabolic state of the cell.

Furthermore, *all-trans* retinoic acid (ATRA) is a vitamin A metabolite involved in the development of tolerogenic immunity (110). This metabolite regulates tolerogenic cytokine production and cell differentiation in monocytes, macrophages, DCs and T cells (111). *In vitro* stimulation of BCG trained monocytes with ATRA inhibits H3K4me3 and induces a strong repressive hallmark (H3K9me) in the promoters of proinflammatory genes such as *tnf*α*, il6, il8, il10*, and *il1ra*. Consequently, the transcription of these genes is silenced, inhibiting the “trained” phenotype (111).

BCG-induced epigenetic reprogramming of monocytes was shown to be able to protect humans against an experimental yellow fever virus (YFV) challenge (112). BCG-vaccinated subjects showed lower viremia after infection with an attenuated YFV vaccine strain. Interestingly, trained immunity induced by BCG was modulated by IL-1β treatment *in vitro*, and cytokine production was increased after vaccination (112). These changes in cytokine secretion were mediated by an increase in H3K4me3 and reduction of H3K9me3 in the promoter regions of the genes *tnf*α*, il6*, and *il1*β (112).

**Figure 3:**
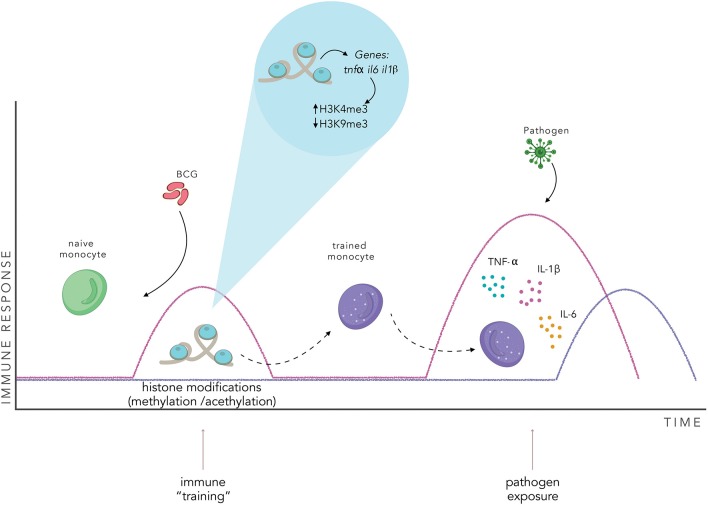
BCG vaccination induces an innate immune training. BCG vaccination activates the innate immune system and induces changes in the pattern of histone modifications of specific genes in innate immune cells. This chromatin rearrangement induces a “trained” state in the cell, enhancing the effectiveness of the innate immune response when exposed to a non-specific pathogen, inducing the secretion of proinflammatory cytokines, such as TNF-α, IL-1β, and IL-6. The pink line represents a trained immune response, the purple line represents a naïve innate immune response.

The genetic reprogramming described above could be implicated in the generation of memory-like innate immune cells (94). In this context, after BCG vaccination, innate immune cells, such as monocytes would undergo a series of chromatin modifications (84, 86, 96). These chromatin rearrangements would lead to a “trained phenotype” that would generate an enhanced innate response when exposed to any non-specific pathogen (Figure 3). Interestingly, chromatin rearrangements induced by BCG vaccination can reprogram bone marrow progenitors, stimulating myelopoiesis, and generating trained immune cells with a higher capacity to protect against a wide variety of pathogens (113, 114). These characteristics of trained immunity suggest that innate immune cells could be a different target for vaccination. As reviewed by Khader et al., vaccination against *M. tuberculosis* with BCG could be directed to the generation of trained hematopoietic progenitors and, in combination with classic vaccination for adaptive immunity generation, generate a greater and more effective immune response (115).

## Recombinant BCG Vaccines

BCG has been considered a good expression vector for recombinant antigens due to several advantages. First, BCG administration is safe for neonates, infants, and adults. Second, BCG doses are relatively easy and non-expensive to produce, allowing for mass-production, and furthermore, it is temperature stable (45). Finally, BCG acts as auto-adjuvant and induces innate and adaptive immune responses (44). Several recombinant BCG (rBCG) strains that express heterologous antigens of different pathogens have been developed and tested since 1991 (25–34). These rBCGs formulations are excellent vaccine candidates, due to the auto-adjuvant characteristics conferred by BCG antigens.

In a mouse model of measles virus (MV) infection, rBCG expressing a nucleocapsid (N) protein of MV significantly reduced viral titers in brain homogenates and mortality due to measles-induced encephalitis (26). Splenocytes from vaccinated mice showed stronger proliferation of antigen-specific T cells in response to MV *in vitro* and an increase of serum MV-specific antibodies as compared to BCG-WT-vaccinated animals (26). This recombinant vaccine was later tested in infant rhesus macaques challenged with an intranasal inoculation of MV (116). Specifically, a total of eight newborn rhesus macaques received the rBCG-MV-N vaccine with no adverse effects, thereby demonstrating its safety. Moreover, rBCG-MV-N vaccination did not induce an increase in antigen-specific antibody titers or expansion of B cell follicles in lymph nodes. The determination of nasopharyngeal viral loads did not show significative differences in the vaccinated groups. Despite this, rBCG-MV-N vaccinated monkeys showed reduced lung pathology after viral challenge in comparison to those vaccinated with WT-BCG and paracortical hyperplasia in lymph nodes, suggesting that protection was mediated by specific T cells (116). Although vaccination with this rBCG was not able to prevent systemic infection, the reduction of lung pathology may prevent MV-associated deaths.

*Toxoplasma gondii* (*T. gondii*) is an intracellular protozoa parasite that infects a variety of warm-blooded animals and is one of the most prevalent human infections worldwide (117). Most immunocompetent humans do not develop clinical signs after acquired infection, but immunocompromised individuals are at risk of developing serious complications, including fatal ones (117). A rBCG expressing rhoptry protein 2 (ROP2), which is a protein of the *T. gondii* involved in host cell invasion induces antigen-specific immune responses in mice (27). Vaccination of mice with a rBCG expressing ROP2 induced the production of specific antibodies, which were detected in serum and induced cellular immune responses (27). Besides, delayed mortality was seen after infection with *T. gondii* in immunized mice (27).

*Bordetella pertussis* is a gram-negative coccobacillus that causes whooping cough (118). This disease causes serious complications including secondary bacterial pneumonia, apnea, bradycardia, pulmonary hypertension, and even death in infants younger than 6 months old (118). An rBCG expressing the S1 subunit of the *B. pertussis* toxin (dPT) was developed and tested as a vaccine in animals (119). Vaccination of mice showed that this vaccine was able to induce the secretion of antigen-specific antibodies and protect mice against a challenge with a lethal dose of B*. pertussis* (119–121). Stimulation of splenocytes of immunized mice with dPT antigen showed an increased capacity to secrete IFN-γ compared to the non-immunized group. Vaccination of 5-day old mice with the rBCG-S1PT vaccine protected them after a lethal dose challenge with *B. pertussis*, showing 100% survival, while non-vaccinated mice had 0% survival 8 days after challenge (121).

Human immunodeficiency virus (HIV) causes acquired immune deficiency syndrome (AIDS) in humans. According to the WHO, this syndrome caused 1.2 million deaths globally in 2016 (122). There is no effective vaccine preventing infection with this virus. Yet, there are many groups all over the world working on the development of an effective vaccine against this virus (28, 29, 31, 45, 123–127). The development of rBCGs expressing HIV antigens has been considered an interesting immunization strategy for a vaccine against HIV. An rBCG expressing the Env protein of the viral capsid of HIV has been shown to develop T_H1_ responses in mice (28). However, it was unable to induce the production of HIV-specific antibodies (28). An rBCG expressing viral antigens used in combination with a booster of viral vectors was also able to induce HIV-specific T cell responses in mice, which was characterized by the secretion of IFN-γ and a T_H1_ polarization (29). rBCG-HIVA, a recombinant BCG that expresses the H and P epitopes of the Env protein and viral polymerase respectively, has been shown to induce the activation of HIV-specific T cells in mice (30). This vaccine in combination with a recombinant viral vector induced robust T cell responses against HIV and *M. tuberculosis* (31). Although these are promising preclinical results, clinical trials must be done to evaluate their efficacy and protection in humans.

The human metapneumovirus (hMPV) is the second major cause of acute lower respiratory tract infections in children and the elderly. This viral infection induces inflammation and disruption of the lung architecture, causing bronchiolitis and pneumonia (128). There is no effective vaccine available to prevent infection with hMPV. However, a novel vaccine has been developed with a rBCG expressing the phosphoprotein (P) protein of hMPV (rBCG-P-hMPV) (32). This rBCG-P-hMPV formulation induces a humoral response against hMPV and can induce viral neutralization and confer protection against hMPV-infection, lowering the amounts of viral particles in the lungs in vaccinated mice (32, 129). Vaccination with rBCG-P-hMPV also reduced T cell infiltration and tissue damage in a mouse model of infection with hMPV, activating a T_H1_-type response and preventing the development of the disease (34).

Another relevant respiratory pathogen is the human orthopneumovirus (previously named human respiratory syncytial virus, hRSV), which is one of the leading causes of acute lower respiratory tract infections in the world (130). In 2015, hRSV caused over 33 million episodes of acute lower respiratory tract infections worldwide, being one of the major causes of hospitalization in children under 5 years of age (131). A rBCG vaccine has been developed expressing the nucleoprotein (N) of the hRSV (rBCG-N-hRSV) (32). In a mice preclinical model, the immunization with rBCG-N-hRSV confers protection against hRSV challenge, reducing both clinical pathology and neutrophil infiltration in the lungs (129). Noteworthy, this vaccine induces the secretion of viral-specific antibodies with neutralizing activity (32), which correlates with lower amounts of viral titers in the lungs in vaccinated mice (129). The rBCG-N-hRSV vaccine has been formulated under good manufacture practices (cGMP) and shown to maintain its promising results in pre-clinical trials. Immunization of mice with rBCG-N-hRSV induces a T_H1_/T_H17_ memory response that was capable of mediating virus clearance and avoiding lung damage (129).

Even though these rBCG strains have shown promising results and protection against their target pathogens, no reports of cross-protection against unrelated pathogens have been published yet. Also, it remains unclear if they maintain the benefits of cross-protection granted by the wild-type strain. Nevertheless, it appears that the trained immunity induced by BCG vaccination could represent additional protection against the pathogen, whose antigen must be expressed by the rBCG. Interestingly, several reports have shown that there is some degree of protection induced by WT-BCG vaccination against other pathogens (95, 96, 106, 112, 132). In the case of *T. gondii*, the determination of IFN-γ secretion by splenocytes stimulated with recombinant ROP2 showed an increased capacity of cells from mice vaccinated with WT-BCG compared to non-vaccinated animals (27), suggesting non-specific protection by WT-BCG against toxoplasmosis. For *B. pertussis*, it is worth noting that splenocytes obtained from mice vaccinated with WT-BCG showed increased IFN-γ secretion after stimulation with dPT as compared to the non-immunized group (121). Surprisingly, WT-BCG vaccinated mice exhibited 80% of survival after a lethal dose challenge, suggesting that the non-specific protection induced by this vaccine can exhibit a protective effect on *B. pertussis* infections and consequently against whooping cough (121). Also, WT-BCG decreases some disease parameters in the mouse models for hRSV and hMPV infection (32). However, the non-specific protection induced by the wild-type vaccine was lower compared to the protection induced by the rBCG expressing hRSV and hMPV antigens, respectively. Mice immunized with the WT-BCG showed intermediate values of viral gene copies and infiltrating neutrophils in the lungs between those that were not immunized and those immunized with the specific rBCG vaccines (rBCG-N-hRSV, and rBCG-P-hMPV) (32). These results support the existence of trained immunity as a consequence of BCG vaccination. The development of a trained innate immune response after rBCG vaccination might be an advantageous scenario for preventing infections, due to the enhanced immune response elicited after immunization against homologous and heterologous pathogens.

## Discussion and Future Challenges

The BCG vaccine has been used in humans for almost a 100 years, proving its immunogenicity and safety. However, it is still unclear whether recombinant BCGs can induce trained immunity after vaccination. Furthermore, the use of the BCG vaccine as a vector for the development of novel recombinant vaccines displays a series of advantages. It is stable, has a low cost of production and acts as an auto-adjuvant, promoting the generation of T_H1_ phenotypes in CD4^+^ T cells that secrete high levels of IFN-γ and that are active against intracellular pathogens. Also, it generates CD8^+^ T cell activation that mediates a cytotoxic response. In addition to protection against M. *tuberculosis*, a characteristic that draws much attention to this vaccine is its effect on decreasing infant mortality in a non-specific manner. This characteristic gives this vaccine a great advantage over other vaccines that confer protection only against the pathogen for which they were developed. Related to this, it was recently described that BCG induces a memory phenotype in innate immune cells, a phenomenon that is known as “trained immunity.” Trained immunity confers non-specific protection against different pathogens, inducing upregulation of PPRs and the secretion of pro-inflammatory cytokines through epigenetic and metabolic reprogramming. This non-specific protection represents a great advantage when used in newborns since they still do not have a complete development of the adaptive immune system and have not been exposed to a wide range of harmful pathogens. Due to this, the training of innate immune cells conferred by BCG could be playing fundamental roles in helping the vaccinated individuals to respond to a wide variety of pathogens.

Trained immunity induced by BCG vaccination can be considered as a potential approach for improving vaccine development and effectiveness, due to the capacity to promote non-specific stimulation of PRRs in innate immune cells. Activation of these cells may contribute to the protection against different pathogens for which no specific vaccine yet exists. As for the case of the influenza A virus, the high mutation rate of this pathogen can impair the effectiveness of highly specific vaccines, thus the broad spectrum of pathogens to which trained immunity response could be a great advantage for the protection to such infectious agents (133, 134). Furthermore, viral infections such as hRSV have been shown to increase the host susceptibility to bacterial infections (135). Thus, vaccines capable of inducing trained immunity could potentially decrease the occurrence of coinfections. Besides, innate immune training strategies could also be applied to children, elderly or immunocompromised individuals that are unable to developing T or B cell-based specific immune responses (136–141). For these cases, the priming of the innate immune response could lead to a better immune response in cases of infections with a broad spectrum of pathogens, as trained immunity has been shown to respond well to viral, bacterial and fungal infections (96, 112, 114).

Even though BCG has been considered a good immunogenic vector, some factors need to be considered when using this bacterium to develop recombinant vaccines. One issue associated with recombinant BCG vaccines is the level of expression of the heterologous antigen (142). The selection of the vector is crucial, given that expression of the heterologous antigen can be mediated by integrative or replicative vectors. In the case of replicative vectors, as there can be more than one copy in a single mycobacterium, increasing the expression level of the antigen of interest (143). Despite this, integrative vectors are more stable and can promote a more stable expression of the antigen in time, which can, in turn, lead to a longer-lasting immune response (143). However, replicative vectors may have a risk of horizontal transfer to other bacteria present in the host, reducing the safety of the vaccine (144). As for the case of viral proteins, these pathogens use the host transcription and translation machinery to produce their proteins (145). Therefore, a potential drawback for using rBCG vaccines for viruses is the possibility that the expression of viral proteins by prokaryotic cells could proteins with variant conformations or altered epitopes (145). To address this problem, the most immunogenic peptides of the protein of interest could be identified and then cloned into a BCG to express only these peptides. In this way, the efficacy of the recombinant vaccine may be improved.

If recombinant BCGs can induce trained immunity after vaccination, we could think that trained immunity induced by rBCG could, in combination with the specific response, induce robust protection against the pathogen of interest. Determination of the development of trained immunity as a consequence of vaccination with recombinant BCG could represent another good advantage for this type of vaccine. Indeed, improving innate immunity represents an ideal complement for the cellular and/or humoral responses developed by rBCG.

## Author Contributions

All authors listed have made a substantial, direct and intellectual contribution to the work, and approved it for publication.

### Conflict of Interest

The authors declare that the research was conducted in the absence of any commercial or financial relationships that could be construed as a potential conflict of interest.
